# Reported High Salt Intake Is Associated with Increased Prevalence of Abdominal Aortic Aneurysm and Larger Aortic Diameter in Older Men

**DOI:** 10.1371/journal.pone.0102578

**Published:** 2014-07-18

**Authors:** Jonathan Golledge, Graeme J. Hankey, Bu B. Yeap, Osvaldo P. Almeida, Leon Flicker, Paul E. Norman

**Affiliations:** 1 Queensland Research Centre for Peripheral Vascular Disease, School of Medicine and Dentistry, James Cook University, Townsville, Australia; 2 Department of Vascular and Endovascular Surgery, The Townsville Hospital, Townsville, Australia; 3 School of Medicine and Pharmacology, University of Western Australia, Perth, Australia; 4 Department of Neurology, Royal Perth Hospital, Perth, Australia; 5 Department of Endocrinology, Fremantle Hospital, Fremantle, Australia; 6 School of Psychiatry & Clinical Neurosciences, University of Western Australia, Perth, Australia; 7 WA Centre for Health & Ageing, Centre for Medical Research, Perth, Australia; 8 Department of Psychiatry, Royal Perth Hospital, Perth, Australia; 9 Department of Geriatric Medicine, Royal Perth Hospital, Perth, Australia; 10 School of Surgery, University of Western Australia, Perth, Australia; University Hospital Medical Centre, Germany

## Abstract

**Background:**

Salt intake has been implicated in the pathogenesis of abdominal aortic aneurysm (AAA) through studies in rodent models but not previously studied in humans. The aim of this study was to examine the association between reported addition of salt to food and the prevalence of AAA.

**Methods:**

A risk factor questionnaire which contained a question about salt intake was included as part of a population screening study for AAA in 11742 older men. AAA presence was assessed by abdominal ultrasound imaging using a reproducible protocol.

**Results:**

The prevalence of AAA was 6.9, 8.5 and 8.6% in men who reported adding salt to food never, sometimes and always, respectively, p = 0.005. Addition of salt to food sometimes (odds ratio [OR]: 1.22, 95% confidence interval [CI]: 1.03–1.44) or always (OR: 1.23, 95% CI 1.04–1.47) was independently associated with AAA after adjustment for other risk factors including age, waist-hip ratio, blood pressure, history of hypertension, high cholesterol, angina, diabetes, myocardial infarction and stroke. Salt intake was also independently associated with aortic diameter (beta 0.023, p = 0.012). In men with no prior history of hypertension, high cholesterol, angina, myocardial infarction or stroke (n = 4185), the association between addition of salt to food sometimes (OR: 1.41, 95% CI 0.96–2.08) or always (OR: 1.52, 95% CI 1.04–2.22) and AAA remained evident.

**Conclusion:**

Reported salt intake is associated with AAA in older men. Additional studies are needed to determine whether reducing salt intake would protect against AAA.

## Introduction

High salt intake has been associated with hypertension in some but not all human association studies [1]. Randomized controlled trials suggest that modest reduction in salt intake over periods of 4–52 weeks leads to significant lowering in blood pressure by 3–4 mmHg [2]. Meta-analyses of prospective studies also suggest that high salt intake is associated with greater risk of stroke [3]. Current clinical guidelines and public health policies recommend low salt intake [4]. There have been, however, reports of increased cardiovascular death associated with low salt intake [5], [6] and there is ongoing controversy over the most appropriate amount of salt intake [7].

Population screening studies suggest that history of hypertension is a risk factor for abdominal aortic aneurysm (AAA) [8], and several animal models utilize salt intake as part of an induction regimen to stimulate aneurysm formation within the aorta and at other arterial sites [10]–[13]. A meta-analysis of large surveillance studies also suggests that hypertension increases the risk of AAA rupture [9]. Based on these data, restricting salt intake should reduce the incidence of AAA, although no clinical studies have assessed the association of salt intake with AAA. The aim of this study was to assess the association of reported salt intake with AAA prevalence. In order to examine this aim we utilized data from the Health In Men Study (HIMS).

## Methods

### Study population

HIMS developed from a population-based randomized trial of screening for AAA conducted in Perth, Western Australia between 1996 and 1999 [14], [15]. The aim of the trial was to assess whether screening reduced mortality from AAA. Eligible men were identified from the electoral roll, with enrolment to vote being compulsory for all Australian adults. Based on a sample size calculation, 41000 men who were resident in Perth and were expected to be aged 65–79 years at the projected mid-point of screening were identified and 19352 were invited, with 12203 (63.1%) attending baseline screening. Ethics approval was obtained from the University of Western Australia Ethics Committee and all men provided written informed consent to participation in the study.

### Assessment of recruited men

Each man was invited to complete a questionnaire assessing aspects of history and lifestyle relevant to AAA and cardiovascular disease including: smoking history; history of diagnosis of high blood pressure, angina, heart attack, stroke, diabetes and high cholesterol; history of treatment for high blood pressure, angina and diabetes; frequency of eating meat (≥6 times/week, 3–5/week, 1–2/week, <1/week or never) and hours of non-vigorous exercise (none, ≤2 hours/ week, >2–4 hours/ week, >4–6 hours/ week or >6 hours/ week). Salt intake was assessed with the following question ‘Do you add salt to your food?’ with three possible answers: (a) rarely or never, (b) sometimes, (c) almost always or always. Waist and hip circumference were measured in accordance with guidelines of the International Society for the Advancement of Kinanthropometry [16]. Blood pressure was measured in line with the guidelines of the British Hypertension Society [17]. Subjects were seated in a quiet room and measurements started after a 10 minute rest period. All measurements were performed in the left arm at the heart level by a trained research nurse using a mercury sphygmomanometer to the nearest mmHg. Mean blood pressure was calculated from the formula [(2× diastolic) + systolic]/3.

### Assessment of aortic diameter

The greatest transverse and antero-posterior diameter of the infra-renal aorta was measured using a Toshiba Capasee ultrasound machine with a 3.75 MHz probe (Toshiba Australia, North Ryde, NSW). Assessment of intraobserver and interobserver reproducibility in aortic diameter measurement was carried out every 4 months on 10 randomly selected subjects, as previously reported [18]. No significant differences were found between observers with 95% of measurement differences being <3 mm [18]. An AAA was defined by infra-renal aortic diameter ≥30 mm.

### Statistical analysis

The association of reported addition of salt to food with cardiovascular risk factors and AAA was examined using chi-squared and Kruskal Wallis test initially. The association of reported salt intake with AAA was examined using binary logistic regression adjusting for frequency of eating meat, frequency of non-vigorous exercise, age, smoking history, history of diagnosis of high blood pressure, angina, heart attack, stroke, diabetes and high cholesterol, history of treatment for high blood pressure, angina and diabetes, waist to hip ratio and mean blood pressure. A sensitivity analysis was performed to assess the association of reported addition of salt to food with AAA in men in which there was no reported prior history of diagnosis or treatment of hypertension, angina, myocardial infarction, stroke or high cholesterol. The association of maximum infrarenal aortic diameter and salt intake was assessed using linear regression, adjusting for frequency of eating meat, frequency of non-vigorous exercise, age, smoking history, history of diagnosis of high blood pressure, angina, heart attack, stroke, diabetes and high cholesterol, history of treatment for high blood pressure, angina and diabetes, waist to hip ratio and mean blood pressure. Mean blood pressure was used in analyses rather than systolic and diastolic blood pressure in order to minimise collinearity, since the values of the latter measures were highly correlated.

## Results

Complete questionnaire data about salt intake was available in 11742 of the 12203 (96.2%) men attending for screening of AAA and these men formed the basis of the current analysis.

Of the 11742 men 4466 (38.0%), 3787 (32.3%) and 3489 (29.7%) reported adding salt to food never, sometimes and always, respectively. Table 1 illustrates the reported frequency of risk factors for cardiovascular disease in relation to reported salt intake. There was no association between reported salt intake and measured blood pressure. A number of cardiovascular risk factors were inversely associated with reported salt intake except smoking and eating meat which were positively associated with reported salt intake (Table 1). Overall 931 of the 11742 (7.9%) men had an AAA identified on ultrasound imaging. The prevalence of AAA was 6.9, 8.5 and 8.6% in men who reported adding salt to food never, sometimes and always, respectively, p = 0.005 (Table 1). Reported salt intake was independently positively associated with AAA after adjustment for other risk factors including frequency of eating meat, frequency of non-vigorous exercise, age, ever smoking, history of diagnosis of high blood pressure, angina, heart attack, stroke, diabetes and high cholesterol, history of treatment for high blood pressure, angina and diabetes, waist to hip ratio and mean blood pressure (Table 2). Men who reported adding salt to their food sometimes had a 1.22 increased odds (95% confidence interval 1.03–1.44) of having an AAA and men who reported adding salt to their food always had a 1.23 increased odds (95% confidence interval 1.04–1.47) of having an AAA. Other risk factors associated with AAA included older age, ever smoking, past history of myocardial infarction, past history of high cholesterol, past treatment for hypertension, past history of angina, past history of stroke, waist to hip ratio and mean blood pressure (Table 2).

**Table 1:** Association of risk factors with reported salt intake in 11742 men.

Reported salt addition to food	Rarely or never	Sometimes	Almost always or always	P value
Number	4466	3787	3489	
Age (years)	71.4±4.3	71.7±4.4	71.6±4.3	0.006
Past history of hypertension	1959 (43.9%)	1499 (39.6%)	1229 (35.2%)	<0.001
Past treatment for hypertension	1749 (39.2%)	1366 (36.1%)	1087 (31.2%)	<0.001
Past history of angina	913 (20.4%)	681 (18.0%)	589 (16.9%)	<0.001
Past treatment for angina	428 (9.6%)	372 (9.8%)	320 (9.2%)	0.634
Past history of myocardial infarction	749 (16.8%)	532 (14.0%)	430 (12.3%)	<0.001
Past history of stroke	381 (8.5%)	263 (6.9%)	259 (7.4%)	0.021
Past history of diabetes	606 (13.6%)	439 (11.6%)	340 (9.7%)	<0.001
Past treatment for diabetes	582 (13.0%)	425 (11.2%)	326 (9.3%)	<0.001
Past history of high cholesterol	1535 (34.4%)	1209 (31.9%)	918 (26.3%)	<0.001
Ever smoker	2936 (65.7%)	2708 (71.5%)	2693 (77.2%)	<0.001
Eat meat (times per week)				<0.001
≥6	1056 (23.6%)	995 (26.3%)	1336 (38.3%)	
3–5	1995 (44.7%)	1805 (47.7%)	1516 (43.5%)	
1–2	1050 (23.5%)	779 (20.6%)	510 (14.6%)	
<1	240 (5.4%)	169 (4.4%)	94 (2.7%)	
Never	125 (2.8%)	39 (1.0%)	33 (0.9%)	
Non-vigorous exercise (hours per week)				<0.001
None	1419 (31.8%)	1283 (33.9%)	1420 (40.7%)	
≤2	660 (14.8%)	548 (14.5%)	433 (12.4%)	
>2–4	913 (20.4%)	720 (19.0%)	588 (16.9%)	
>4–6	488 (10.9%)	394 (10.4%)	327 (9.4%)	
>6	986 (22.1%)	842 (22.2%)	721 (20.7%)	
WHR*	0.95±0.06	0.96±0.06	0.97±0.06	<0.001
Systolic blood pressure (mmHg)	157±21	157±21	157±21	0.479
Diastolic blood pressure (mmHg)	90±12	90±12	90±12	0.824
Mean blood pressure (mmHg)	112±13	112±14	112±14	0.941
Aortic diameter (mm)	22.7±5.2	23.0±5.7	23.2±5.9	0.002
AAA	308 (6.9%)	322 (8.5%)	301 (8.6%)	0.005

Nominal variables are presented as numbers (%) and compared by chi-squared. Continuous variables are presented as mean (± standard deviation) and compared by Kruskal Wallis test.

*WHR data missing on 6 men. WHR =  Waist to hip ratio; AAA =  Abdominal aortic aneurysm.

**Table 2:** Independent association of reported salt intake with prevalent AAA in 11742 older men.

Characteristic	Odds ratio	95% CI	P value
Reported salt addition to food:			
Rare	1.00	Reference	
Sometimes	1.22	1.03–1.44	0.021
Always	1.23	1.04–1.47	0.018
Age (per 4 years)*	1.33	1.25–1.42	<0.001
Past history of hypertension	1.01	0.81–1.25	0.955
Past treatment for hypertension	1.41	1.14–1.74	0.002
Past history of angina	1.27	1.03–1.56	0.027
Past treatment for angina	0.83	0.65–1.07	0.149
Past history of myocardial infarction	1.76	1.46–2.13	<0.001
Past history of stroke	1.26	1.01–1.57	0.040
Past history of diabetes	0.65	0.32–1.32	0.232
Past treatment for diabetes	1.16	0.57–2.36	0.689
Past history of high cholesterol	1.29	1.11–1.50	0.001
Ever smoker	2.57	2.12–3.13	<0.001
WHR (per 0.06)*	1.12	1.05–1.20	0.001
Mean blood pressure (per 14 mmHg)*	1.09	1.01–1.17	<0.001
Eat meat (times per week)			
≥6	0.86	0.50–1.47	0.855
3–5	0.96	0.56–1.64	0.880
1–2	0.86	0.50–1.48	0.582
<1	0.85	0.46–1.59	0.619
Never	1.00	Reference	
Non-vigorous exercise (hours per week)			
None	1.00	Reference	
≤2	1.02	0.83–1.26	0.830
>2–4	0.92	0.76–1.12	0.411
>4–6	0.87	0.68–1.11	0.262
>6	0.91	0.75–1.10	0.324

Men with the risk factor were compared to subjects without the risk factor. All variables shown were included in the multivariate model.

*Approximate standard deviation. WHR =  Waist to hip ratio; AAA =  Abdominal aortic aneurysm.

Men who reported adding salt to food never, sometimes and always had a mean maximum infrarenal aortic diameter of 22.7±5.2, 23.0±5.7 and 23.2±5.9 mm, respectively, p = 0.002 (Table 1). Salt intake was independently associated with aortic diameter after adjusting for other risk factors, beta 0.023, p = 0.012, Table 3.

**Table 3:** Independent association of reported salt intake with aortic diameter in 11742 older men.

Characteristic	Standardised coefficients (beta)	t value	P value
Reported salt addition to food	0.023	2.504	0.012
Age	0.096	10.417	<0.001
Past history of hypertension	−0.006	0.449	0.653
Past treatment for hypertension	0.047	3.317	0.001
Past history of angina	0.022	1.830	0.067
Past treatment for angina	0.004	0.330	0.741
Past history of myocardial infarction	0.066	6.304	<0.001
Past history of stroke	0.010	1.042	0.298
Past history of diabetes	−0.025	0.849	0.396
Past treatment for diabetes	−0.015	0.529	0.597
Past history of high cholesterol	0.010	1.011	0.312
Ever smoker	0.091	9.952	<0.001
WHR	0.061	6.469	<0.001
Mean blood pressure	0.046	4.807	<0.001
Eat meat	−0.003	0.307	0.759
Non-vigorous exercise	−0.011	1.251	0.211

All variables shown were included in the multivariate model. WHR =  Waist to hip ratio.

Given the negative association between high cholesterol, hypertension, angina, myocardial infarction and stroke with addition of salt to food we performed a sensitivity analysis excluding men with these diagnoses. These exclusions removed 7557 men, leaving 4185 men of whom 199 had an AAA for further assessment. In this sub-group the prevalence of AAA in men reporting adding salt to food rarely, sometimes or always was 45 of 1384 (3.3%), 71 of 1348 (5.3%) and 83 of 1453 (5.7%), respectively. After adjustment for other risk factors adding salt to food sometimes or always were associated with AAA (odds ratios 1.41, 95% confidence interval 0.96–2.08, and 1.52, 95% confidence interval 1.04–2.22, respectively, Table 4).

**Table 4:** Independent association of reported salt intake with prevalent AAA in 4185 older men with no history of high cholesterol, hypertension, angina, myocardial infarction or stroke.

Characteristic	Odds ratio	95% CI	P value
Reported salt addition to food:			
Rare	1.00	Reference	
Sometimes	1.41	0.96–2.08	0.083
Always	1.52	1.04–2.22	0.032
Age (per 4 years)*	1.49	1.31–1.70	<0.001
Past history of diabetes	0.90	0.18–4.55	0.897
Past treatment for diabetes	0.99	0.18–5.28	0.986
Ever smoker	2.52	1.69–3.76	<0.001
WHR (per 0.06)*	1.25	1.08–1.43	0.002
Eat meat (times per week)			
≥6	0.61	0.18–2.04	0.420
3–5	0.81	0.24–2.69	0.731
1–2	0.71	0.21–2.41	0.578
<1	0.20	0.03–1.23	0.082
Never	1.00	Reference	
Non-vigorous exercise (hours per week)			
None	1.00	Reference	
≤2	0.98	0.62–1.55	0.979
>2–4	0.84	0.55–1.30	0.439
>4–6	0.89	0.52–1.51	0.665
>6	0.88	0.59–1.30	0.504
Mean blood pressure (per 14 mmHg)*	1.11	0.95–1.30	0.204

Men with the risk factor were compared to subjects without the risk factor. All variables shown were included in the multivariate model.

*Approximate standard deviation. WHR =  Waist to hip ratio; AAA =  Abdominal aortic aneurysm.

## Discussion

The main findings from this study were that sometimes or always adding salt to food was associated with increased prevalence of AAA and larger infrarenal aortic diameter in older men. The reliability of this association is supported by the large number of men examined (11742), the demonstrated reproducible method of aortic imaging used, the adjustment for potential confounding risk factors, and the consistency of the association when men with previous cardiovascular disease or relevant risk factors were excluded.

We believe this to be the first study to examine the association of reported salt intake with aortic aneurysm in humans. The role of high salt intake in aortic aneurysm formation has been previously examined in three rodent model studies [10]–[12]. Nishijo and colleagues examined the effect of adding 1% sodium chloride to the drinking water of transgenic mice that overproduced angiotensin II [12]. They reported that mice receiving salt loading developed thoracic and abdominal aortic aneurysms which ruptured in 67% of animals during 30 days of salt administration. Nishijo et al. reported that salt loading stimulated an increase in drinking volume and plasma atrial natriuretic peptide associated with loss of aortic vascular smooth muscle cells [12]. Kanematsu and colleagues administered the mineralocorticoid deoxycorticosterone acetate, 1% salt and a lysyl oxidase inhibitor, which blocks cross-linking of elastin and collagen, to male C57BL/6J mice. This regimen induced thoracic and abdominal aortic aneurysms associated with hypertension, aortic wall inflammation and disruption of elastic laminae [11]. Finally, Liu and colleagues examined the effect of the administration of the mineralocorticoid receptor agonists deoxycorticosterone acetate and aldosterone in addition to 1% salt. They reported that either of these mineralocorticoid receptor agonists when administered with salt but not alone induced thoracic and abdominal aortic aneurysms [10]. Salt when administered with a mineralocorticoid receptor agonist induced aortic elastin degradation, elevated matrix metalloproteinase activity, vascular smooth muscle cell apoptosis, macrophage and neutrophil infiltration, and upregulation of a range of inflammatory and oxidative stress markers. Histological evidence of aortic dissection was also demonstrated in 40% of the mice. While aneurysm induction was associated with hypertension, the rise in blood pressure induced was not correlated with the size or incidence of aneurysm development [10], [19]. Our study provides the first evidence in older men, who are most at risk of AAA, that, similar to rodents, salt intake is associated with AAA.

Salt administration has also been used to induce cerebral aneurysm formation in rodents [13]. The common feature of all these rodent model studies appears to be the administration of salt along with activation of a part of the renin-angiotensin-aldosterone system [19]. Raised blood pressure is another common feature of these models however lowering blood pressure does not necessarily inhibit aneurysm formation [10], [11], [19]. These findings are in keeping with our observation that higher salt intake was associated with AAA independently of a history of hypertension or treatment for hypertension and measured mean blood pressure. High salt intake has been demonstrated to increase a range of neural, endocrine and renal changes which could promote cardiovascular disease including AAA (see previous reviews 1, 19). These changes promote aortic inflammation, angiogenesis, loss of aortic elasticity and oxidative stress, which are all implicated in AAA pathogenesis [1], [8], [20], [21], [22].

A number of possible limitations of this study should be considered including measurement error, reverse causality and residual confounding. Firstly, our assessment of salt intake was limited to a simple questionnaire in which we asked whether salt was added to food never or rarely, sometimes, almost always or always. More sophisticated assessment methods, such as measured of 24-hour urinary sodium excretion, were not used. This approach may have introduced measurement error. It is however accepted that even biochemical methods of estimating salt intake are open to measurement error [23]. Furthermore, self-reported dietary intake of salt has been found to be reflective of 24 hour urinary sodium excretion [24], suggesting that self-report is a valid measure of salt intake. Secondly, this study was a cross-sectional human association study. It is not possible to conclude that the association between self-reported high salt intake and AAA is causative. The direct role of salt in AAA development could only be established by a randomized controlled trial of at risk individuals in which the effect of administering different amounts of salt was compared. Such a trial would require a large number of subjects and extended follow-up in order to assess the efficacy of salt restriction on AAA incidence. Thirdly, the mean aortic diameter differences between patients reporting different levels of salt intake were small and within the measurement error of aortic imaging. While we established the reproducibility of the ultrasound imaging during the course of the study it is possible, although we believe unlikely, that measure error may have confounded our findings. Fourthly, since we only studied men we can make no comment on how our findings relate to women. Finally we may have failed to adjust for some confounding factors. The current study included a large number of men and used adjustment for recognized confounding factors such as age, hypertension, high cholesterol, coronary heart disease and stroke. It is possible that other confounding factor, such as prescription of diuretics, which we were not able to assess may have contributed to our finding.

In conclusion the current study identifies for the first time a positive association between reported high salt intake and the prevalence of AAA in older men. This finding, along with rodent model data, suggests that high salt intake increases the risk of AAA development independent of other known causal factors. This information fits with data from other cardiovascular diseases and suggests that salt intake should be restricted. What would represent a safe amount of salt intake is yet to be established.
